# PSY–PSYR peptide–receptor pairs control the trade-off between plant growth and stress response

**DOI:** 10.1080/15592324.2023.2260638

**Published:** 2023-09-22

**Authors:** Quy Thi Cam Nguyen, Jungmook Kim

**Affiliations:** a Department of Integrative Food, Bioscience and Biotechnology, Chonnam National University, Gwangju, Korea; b Department of Bioenergy Science and Technology, Chonnam National University, Gwangju, Korea

**Keywords:** Leucine-rich repeat-receptor kinase, plant peptide, PSY, PSYR, stress response

## Abstract

Leucine-rich repeat-receptor kinases (LRR-RKs) perceive various endogenous peptide hormones that control plant growth and development. However, the majority of corresponding ligands and their direct ligand-binding receptors have not been identified yet. A recent study demonstrated that three LRR-RK PLANT PEPTIDE CONTAINING SULFATED TYROSINE RECEPTORS (PSYRs) act as ligand-receptors of the PSY family peptides that mediate the trade-off between the optimal plant growth and stress tolerance responses. The genetic, biochemical, and transcriptome analyses suggested that PSYR1, PSYR2, and PSYR3 function as negative regulators of plant growth in the absence of PSY peptides and induce stress tolerance responses, whereas the PSY family peptides repress PSYR signaling, allowing plant growth. This trade-off mechanism between plant growth and stress responses mediated by the PSY–PSYR signaling module allows plants to survive under ever changing environmental stresses.

## Introduction

Signaling peptides act as phytohormones that control plant growth, development, and stress responses via cell–to–cell and long-distance communication networks. Peptides are encoded by gene families. It is predicted that *Arabidopsis* contains > 1000 peptides. Plant peptides bind to membrane-embedded leucine-rich repeat-receptor kinases (LRR-RKs), inducing the activation of cellular signaling.^1^ In the *Arabidopsis* genome, >200 LRR-RKs have been identified so far.^2,3^ LRR-RKs play essential roles in plant development, such as cell elongation, cell division, meristem development, and vascular patterning, and biotic and abiotic stress responses.^4,5^ They contain an extracellular LRR domain, a single transmembrane region, and a cytosolic kinase domain. Their kinase domain causes auto- and trans-phosphorylation with co-receptors such as SOMATIC EMBRYOGENESIS RECEPTOR KINASES (SERKs), associated with LRR-RKs, in response to their cognate ligand peptides to activate downstream signaling components.^5^

PLANT PEPTIDE CONTAINING SULFATED TYROSINE 1 (PSY1) is an 18-amino-acid sulfated and glycosylated peptide that promotes cell expansion and differentiation in the elongation zone of roots.^6–8^ The PSY1 RECEPTOR (PSY1R) activates the plasma membrane H^+^-ATPases (AHAs) by interacting with and phosphorylating AHAs for cell expansion and proliferation.^9,10^ RaxX, a PSY1-like sulfated peptide, is produced by the biotrophic pathogen *Xanthomonas oryzae pv. oryzae* (*Xoo*) in rice and binds the host PSY1R to activate its downstream signaling.^11,12^ RaxX triggers the immune response by directly binding to immune receptor XA21.^13;14^ However, the fundamental roles of PSY signaling during plant growth and development are still unknown. Moreover, the direct receptors of PSY family peptides have not yet been identified.

## PSY family peptides are directly recognized by three LRR-RKs

A recent study reported that three orphan LRR-RKs act as direct ligand-receptors for the PSY family peptides and mediate the trade-off between plant growth and stress response.^15^ By conducting nano-liquid chromatography-coupled tandem mass spectrometry (LC-MS/MS) analyses, Ogawa-Ohnishi et al. identified the secreted and mature forms of PSY5, PSY6, and PSY8 peptides exhibiting sequence similarity with PSY1. Using photo-affinity labeling experiments, the authors showed that three related LRR-RKs, designated as PSYR1, PSYR2, and PSYR3, directly interact with PSY5 and PSY6. Furthermore, RaxX16 and RaxX21, synthetic sulfated RaxX peptides, also bind to PSYR2 and PSYR3. These results indicated that PSYRs recognize both PSY family peptides and RaxX peptides.

## PSY–PSYR signaling mediates trade-off between growth and stress response

**Figure 1.**
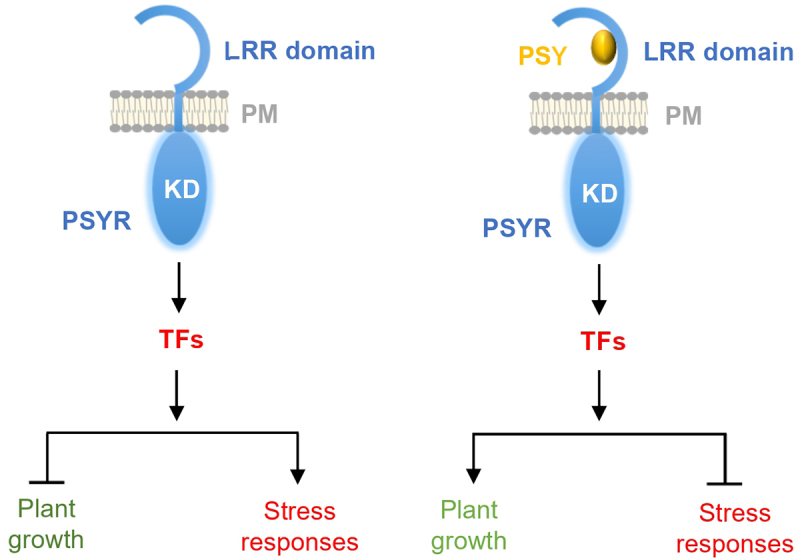
Model of PSY-PSYR module regulating switching between plant growth and stress response. In the absence of PSY peptides, PSYRs induce stress tolerance responses but suppress plant growth (left panel). In the presence of PSY peptides, PSYRs enable plant growth but restrict stress response (right panel). KD, kinase domain; LRR, leucine-rich repeat domain; PM, plasma membrane; PSY, PLANT PEPTIDE CONTAINING SULFATED TYROSINE 1; PSYR, PSY RECEPTOR; TF, transcription factor.

The tyrosylprotein sulfotransferase *tpst-1* mutant is deficient in the biosynthesis of all tyrosine-sulfated peptides and serves as a substitute for the poly-mutant of sulfated peptide hormones.^16–18^ To understand the molecular mechanism underlying PSY–PSYR signaling, phenotypes of *tpst-1* and the receptor triple mutant *psyr1,2,3* were analyzed.^15^ The PSY5 peptide was effective in rescuing the root growth defects in *tpst-1* and thus selected as a representative of the PSY family. However, the root growth of *psyr1,2,3* mutants were enhanced. PSY5 treatment had no effect on the root growth of *psyr1,2,3*. To confirm the receptor mutant phenotype, the *tpst-1/psyr1,2,3* quadruple mutant was generated. From the phenotype analyses of the *tpst-1/psyr1,2,3* quadruple mutant, it was concluded that PSYR1, PSYR2, and PSYR3 function as redundant negative regulators of plant growth in the absence of the PSY peptide, whereas the PSY family peptides repress PSYR signaling, allowing plant growth.^15^ Their data suggested that in the absence of PSY peptides, PSYRs stimulate stress signaling and suppress plant growth, whereas in their presence, stress signaling is inhibited, which allows plant growth (Figure 1). Transcriptome analyses showed that *tpst-1/psyr1,2,3* and PSY5-treated *tpst-1* plants have highly similar transcriptional profiles.^15^ Unexpectedly, the *psyr1,2,3* mutant displays a constitutive PSY response phenotype. They identified 26 transcription factor genes mostly conferring biotic and abiotic stress tolerance that are downregulated by PSY5 treatment in *tpst-1*. Therefore, the growth-promoting effect of PSY family peptides may be a trade-off between growth and stress tolerance. Further, they showed that PSYRs are essential for plant resistance against multiple biotic and abiotic stresses, such as high salinity, high temperature, and pathogen invasion.

## Concluding remarks and future perspectives

Matsubayashi and his colleagues provided compelling evidence that three LRR-RKs, PSYR1, PSYR2, and PSYR3, act as ligand-receptors of PSY family peptides and mediate switching between two opposing pathways: plant growth and stress response.^15^ The results of their study demonstrate a previously unknown cell-to-cell communication pathway for plants to survive under environmental stress conditions. Ligand-deprivation-dependent activation systems induce prophylactic stress responses within viable cell layers adjacent to the damaged tissue sites. This allows optimal plant growth under stressful conditions by balancing stress tolerance with energetic costs. However, the molecular mechanisms responsible this pathway have yet to be identified. For example, the PSY– PSYR signaling pathway, especially that involving the trade-off between growth and stress response, is unknown. Co-immunoprecipitation followed by mass spectrometric analysis showed that PSYR3-GFP does not bind to the previously known co-receptors, irrespective of the PSY5 treatment. The atypical co-receptors and downstream signaling components of the PSY–PSYR module need to be identified. Gain-of-function approaches, such as rapamycin-inducible dimerization of engineered receptor–co-receptor pairs^19^ and constitutive activation of LRR-RK signaling by BAK1-INTERACTING RECEPTOR-LIKE KINASE3 chimera,^20^ could be used to investigate whether the activation of PSYR signaling by the association of SERK co-receptors in plants induce receptor phenotypes and downstream cellular responses. Interestingly, the expression of stress-related 26 transcription factor genes is highly elevated in *tpst-1* compared with that of the wild type, indicating that the PSY–PSYR pathway controls these stress-related multiple transcription factors to mediate the trade-off between stress response and optimal plant growth. It will be of interest to identify target genes of these transcription factors acting downstream of the PSY – PSYR signaling module. PSY1R signaling has been reported to be involved in crosstalk between various phytohormones, including auxin, in plant growth, development, and defense response.^10^ Future studies on PSY – PSYR signaling as a regulator of plant growth, stress responses, and hormone homeostasis may help understand the mechanisms by which plants adapt to ever changing environmental stresses.^10^
